# A Rare Overlap of Serotonin Syndrome and Status Epilepticus Confirmed on Electroencephalogram

**DOI:** 10.7759/cureus.4667

**Published:** 2019-05-15

**Authors:** Binod Wagle, Margaret Finn, Naga Prasuna Vanipenta

**Affiliations:** 1 Department of Neurology, University of Missouri - Kansas City School of Medicine, Truman Medical Center, Kansas City, USA; 2 Department of Neurology, University of Missouri - Kansas City School of Medicine, Kansas City, USA; 3 Department of Psychiatry, University of Missouri - Kansas City School of Medicine, Kansas City, USA

**Keywords:** seizure, serotonin syndrome, depression, depression, status epilepticus, fluoxetine, selective serotonin reuptake inhibitors (ssri), bupropion, electroencephalogram (eeg), anticonvulsant

## Abstract

Serotonin syndrome (SS) is a potentially fatal complication of treatment with various serotonergic agents. It is diagnosed clinically and consists of cognitive, autonomic, and neuromuscular dysfunction. Although serotonin syndrome has been known to induce seizures, there are no reported cases of electroencephalogram (EEG)-documented status epilepticus (SE) associated with serotonin syndrome. We report a case of serotonin syndrome and status epilepticus in a patient thought to have overdosed on both fluoxetine and bupropion in the setting of alcohol intoxication. Our patient required aggressive treatment with various anticonvulsant medications to control status epilepticus and was also treated with cyproheptadine for the serotonin syndrome. This paper will also discuss the contributing factors of fluoxetine and bupropion to this presentation in the context of alcohol intoxication.

## Introduction

Serotonin syndrome (SS) is a potentially life-threatening complication of treatment with serotonergic agents, either from a combination of these agents or from toxicity of a single agent [1-4]. This occurs most commonly due to interactions between selective serotonin re-uptake inhibitors (SSRIs), tricyclic antidepressants, and monoamine oxidase inhibitors [2,3]. Fluoxetine is an SSRI that increases the post-synaptic activity of serotonin and can be used for depression [5]. Munhoz reported a case of SS caused by bupropion [4]. Prior to that report, there were no published cases, although bupropion has been listed as a potential causative agent [4,6]. Bupropion is an atypical antidepressant that has selective re-uptake inhibition of dopamine and norepinephrine with weak actions on serotonin [4,6].

Patients with serotonin syndrome classically exhibit a triad of cognitive, autonomic, and neuromuscular dysfunction [1-3,7]. This can include altered mental status, hyperthermia, agitation, hyperreflexia, clonus, tremor, diaphoresis, mydriasis, and muscular rigidity [1-3,7,8]. Although seizures have been reported with serotonin syndrome, there have been only rare reports of status epilepticus (SE) [1,7,8]. Status epilepticus, an epilepsy emergency, is defined as five minutes or longer of continuous seizure activity or recurrent seizures without recovery between seizures [9,10]. Both status epilepticus and serotonin syndrome require rapid and appropriate treatment, which influences patient prognosis. We report a case of serotonin syndrome and electroencephalogram (EEG)-confirmed status epilepticus in a patient thought to have overdosed on both fluoxetine and bupropion in the setting of alcohol intoxication.

## Case presentation

A 22-year-old patient with a past history significant for depression, anxiety, and alcohol use disorder presented to the emergency department with altered mental status after multiple convulsive seizures. The patient was persistently hypotensive with an initial Glasgow Coma Scale 3/15. Intubation and sedation with propofol were necessary. Naloxone had been administered on route to the emergency department (ED) for concern of opiate overdose. Emergency medical services (EMS) reported that the patient suffered multiple episodes of emesis and convulsions. The patient had no prior history of seizure. Blood alcohol level measured in the emergency department was 189 mg/dl and the urine drug screen was negative. Multiple myoclonic and tonic-clonic movements were observed, indicating recurrent seizures. The patient was loaded with levetiracetam and midazolam.

On examination with sedation held, the patient had no response to noxious stimuli. Pupils were fixed and dilated with absent corneal reflex. Cough reflex was intact. Motor examination revealed bilateral rigidity, most prominent in lower extremities. Deep tendon reflexes were diffusely brisk with bilateral ankle and ocular clonus (spontaneous, rapid but equal horizontal movements of both eyes). Diffuse focal and multi-focal myoclonus was observed and persisted while on sedation.

**Figure 1:**
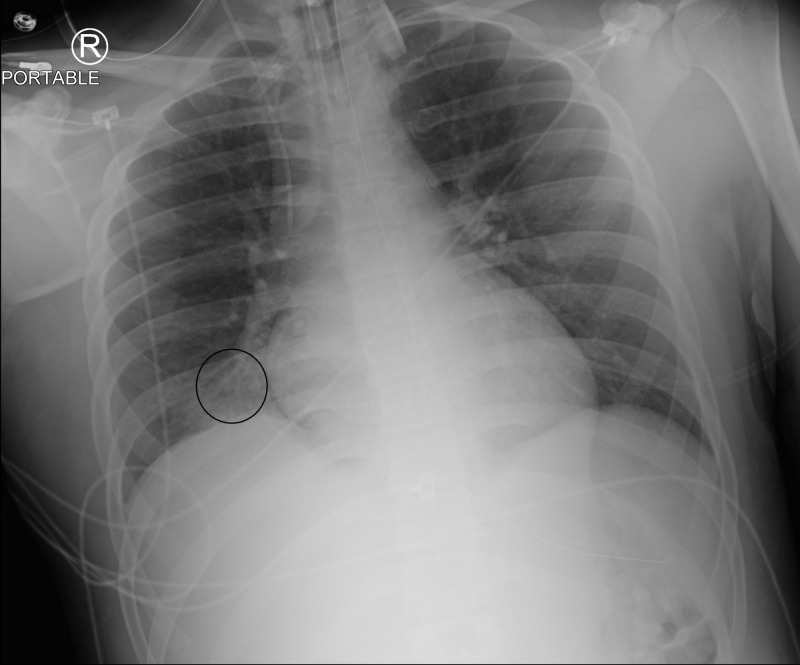
Right lower lobe opacity (marked by circle), concerning for aspiration pneumonia.

Laboratory studies revealed ammonia of 162 umol/L (ref range: 16-60 umol/L), elevated creatinine at 1.54 mg/dl (ref range: 0.9-1.3 mg/dl), lactic acidosis of 18.8 mmol/L (ref range: 0.5-2.2 mmol/L), leukocytosis at 18,500/cmm (ref range 4,300-10,800/cmm), and elevated creatinine kinase at 712 U/L (ref range: 38-234 U/L). Bicarbonate level was critically low at 7 mmol/L (ref range: 22-32 mmol/L) and anion gap was 38 mmol/L (ref range: 10-20 mmol/L). Head computed tomography (CT) showed no acute findings. Chest radiograph (Figure 1) was also obtained and there was an asymmetric increased opacity in the right lower lobe, concerning for aspiration pneumonia.

**Figure 2:**
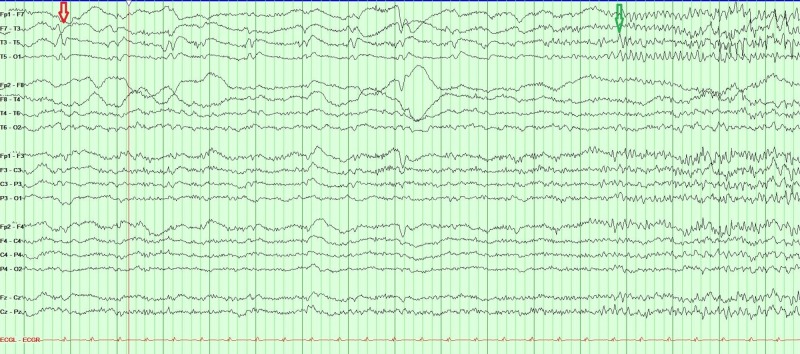
Red arrow showing left temporal sharps. Green arrow showing the onset of a seizure.

**Figure 3:**
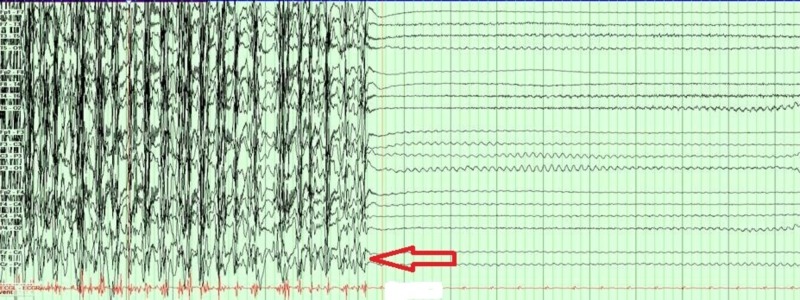
Red arrow showing cessation of a seizure. Video EEG was able to capture 17 similar seizures in the first two hours.

Despite propofol, midazolam and levatiracetam, the patient continued to have tonic-clonic and myoclonic movements, clinically consistent with status epilepticus. Immediate electroencephalogram (EEG) and subsequent continuous video EEG monitoring were performed. This captured epileptiform activity arising from the left temporal area, which evolved into rhythmic high frequency alpha and beta activity and quickly spread to bilateral hemispheres (Figure 2). Following this was the slowing of the rhythmic activity to theta, and 2-3 Hz poly-spikes and waves, then completely suppressed background activity afterward (Figure 3), which corresponded with cessation of tonic-clonic seizure seen on the video. All rhythmic activity lasted 30-40 seconds. Video EEG was able to capture 17 similar events in the first two hours confirming status epilepticus. The patient was loaded with lacosamide in addition to levetiracetam, and propofol and midazolam were further increased with a goal of burst suppression pattern. EEG monitoring did not capture any further electrographic or clinical seizures.

The second day, the patient developed fever up to 104° F. Empiric treatment was started for suspected meningitis, and lumbar puncture was completed with normal results. Initial blood cultures sent from the emergency department were negative. Repeat blood cultures and urinary analysis and culture were sent and reported negative. Chest radiograph was also repeated and was found to be stable.

There was a high suspicion of serotonin syndrome (SS) due to the patient’s mental status, hyperthermia, diffuse rigidity, hyperreflexia, ankle and ocular clonus, and multi-focal myoclonus. Additional history obtained from the family members indicated that bupropion and fluoxetine were home medications and that 20 days’ supply in each prescription bottle was missing. The patient was started on cyproheptadine on the third day for suspected serotonin syndrome. Sedation with propofol and midazolam were maintained and the patient was continued on levetiracetam and lacosamide as anticonvulsant medication.

Magnetic resonance imaging (MRI) obtained on Day 3 was unremarkable. Chest radiograph continued to be stable throughout the stay. Intermittent fevers up to 101.1° F occurred, and repeat blood cultures were negative. Decreasing sedation began on Day 4, the rigidity resolved by Day 6, and the patient was extubated on Day 10 of hospitalization. Cyproheptadine was discontinued the following day. The psychiatry department evaluated the patient prior to discharge with the impression that the overdose was due to a substance-induced impulse without intentional harm. The patient was discharged home on levetiracetam with a normal mental status.

## Discussion

Serotonin syndrome (SS) is a potentially fatal complication that occurs from ingestion of serotonergic drugs, either multiples in combination or from toxic doses [1-4]. It is a clinical diagnosis made using the Hunter Criteria. Classically patients have a triad of cognitive, autonomic, and neuromuscular dysfunction [1,2]. The key components of Hunter criteria are spontaneous clonus, inducible clonus, ocular clonus, tremor and hyperreflexia, hypertonia, and hyperthermia [1,2,7]. Laboratory results show nonspecific changes such as leukocytosis, elevated creatinine kinase, elevated creatinine, or decreased bicarbonate [7]. Complications such as rhabdomyolysis, acute kidney failure, and metabolic acidosis may occur [7]. Treatment includes discontinuation of the causative agents, supportive treatment for autonomic changes, and cyproheptadine [2,7,8].

Serotonin syndrome has been known to induce seizures, but only rare reports have shown an association with status epilepticus (SE) and no known reports have documented SE on EEG in this setting [1,7,8]. SE is classically defined as a seizure over five minutes or recurrent seizure activity without recovery between seizures [9,10]. It is associated with a mortality rate of up to 20% [9,10]. A seizure occurs due to abnormal electrical discharge in the cortex from a hyper-excitable neuron. Adjacent neurons become excited resulting in hyper-synchronization [9]. This hyper-excitability can be caused by an imbalance of excess glutamate, the excitatory neurotransmitter or not enough gamma aminobutyric acid (GABA), the inhibitory neurotransmitter [9]. A lowered seizure threshold may also be contributing factor. It can be lowered by mood modulators such as alcohol, illegal drugs, and medications like bupropion [11]. Alcohol is a major precipitant of SE, although it is not commonly seen with alcohol intoxication [11].

The patient in this case overdosed on both fluoxetine and bupropion. Fluoxetine is an SSRI that increases the post-synaptic activity of serotonin and can be used for depression [12]. The half-life is one week after long-term use [2,7]. It has also been found to potentiate the effects of bupropion [12]. Bupropion is an atypical antidepressant that is a selective re-uptake inhibitor of dopamine and norepinephrine with a minimal effect on serotonin [4,6]. Although considered a causative agent for status epilepticus, that has rarely been proven [4]. Bupropion is known to decrease seizure threshold. The effects of bupropion are dose-dependent [12]. It is also a CYP2D6 inhibitor that could increase effects of fluoxetine [4,12].

The patient in this case fulfilled the Hunter criteria for SS and had SE, with no prior history of seizures [1,7]. There were multiple contributing factors accounting for the SS and SE seen in this patient-particularly, the interactions between fluoxetine and bupropion, the overdosed medications. Bupropion lowers the seizure threshold and is dose-dependent and its effect is also potentiated by the use of fluoxetine [12]. Alcohol intoxication, although not routinely seen with seizures, also lowers the seizure threshold [11]. All of these factors could account for the SE seen in this patient. Bupropion is a CYP2D6 inhibitor that increases the effects of fluoxetine [4]. Accounting for the theory of overdose on top of this metabolism inhibition, this compounds the likelihood of serotonin syndrome (SS). Fluoxetine also has an extended half-life, which explains the delayed presentation of serotonin syndrome and the long duration of symptoms after discontinuation of the serotonergic medications [2,7]. All of these factors contributed to the unique presentation of this patient with serotonin syndrome, status epilepticus, and alcohol intoxication.

## Conclusions

Although serotonin syndrome has been known to induce seizures, there have been no reported cases associated with status epilepticus (SE) recorded on electroencephalogram (EEG) as was seen in our case. There were multiple contributing factors in our patient accounting for the serotonin syndrome and status epilepticus, including the overdose with bupropion and fluoxetine, and the suspected alcohol intoxication. Both conditions, have a clear time-dependent relationship to morbidity and mortality. Our patient required aggressive treatments with various anticonvulsant medications to control status epilepticus and was also treated with cyproheptadine for the serotonin syndrome.

## References

[1] Bosak AR, Skolnik AB (2014). Serotonin syndrome associated with metaxalone overdose. J Med Toxicol.

[2] Buckley NA, Dawson AH, Isbister GK (2014). Serotonin syndrome. BMJ.

[3] Mason PJ, Morris VA, Balcezak TJ (2000). Serotonin syndrome. Presentation of 2 cases and review of the literature. Medicine (Baltimore).

[4] Munhoz RP (2004). Serotonin syndrome induced by a combination of bupropion and SSRIs. Clin Neuropharmacol.

[5] Wood DM, Rajalingam Y, Greene SL, Morgan PE, Gerrie D, Jones AL, Dargan PI (2007). Status epilepticus following intentional overdose of fluvoxamine: a case report with serum fluvoxamine concentration. Clinical Toxicology.

[6] Morazin F, Lumbroso A, Harry P, Blaise M, Turcant A, Montravers P, Gauzit R (2009). Cardiogenic shock and status epilepticus after massive buproprion overdose. Clin Toxicol (Phila).

[7] Volpi-Abadie J, Kaye AM, Kaye AD (2013). Serotonin syndrome. Ochsner J.

[8] Prakash S, Patel V, Kakked S, Patel I, Yadav R (2015). Mild serotonin syndrome: a report of 12 cases. Ann Indian Acad Neurol.

[9] Khoujah D, Abraham M (2016). Status epilepticus. Emerg Med Clin North Am.

[10] Seinfeld S, Goodkin HP, Shinnar S (2016). Status epilepticus. Cold Spring Harb Perspect Med.

[11] Hillbom M, Pieninkeroinen I, Leone M (2003). Seizures in alcohol-dependent patients: epidemiology, pathophysiology and management. CNS Drugs.

[12] Li SX, Perry KW, Wong DT (2002). Influence of fluoxetine on the ability of bupropion to modulate extracellular dopamine and norepinephrine concentrations in three mesocorticolimbic areas of rats. Neuropharmacology.

